# Phylogenetic and taxonomic study of the complete mitochondrial genome of *Spodoptera frugiperda*

**DOI:** 10.1080/23802359.2019.1644546

**Published:** 2019-07-25

**Authors:** Tingting Liu, Zhaofei Li

**Affiliations:** College of Plant Protection, Northwest A&F University, Shaanxi, Yangling, China

**Keywords:** *Spodoptera frugiperda*, the mitochondrial genome, taxonomic analysis, phylogenetic analysis

## Abstract

*Spodoptera frugiperda* is a polifagous insect of major economic impact in the world. In this study, we assembled and annotated the complete mitochondrial genome of *S. frugiperda*. The mitochondrial genome length is 15,353 bp, containing 13 protein-coding genes (PCGs), 22 transfer RNA genes (tRNA), 2 ribosome RNA genes (rRNA), and 1 D-loop control regions. The overall nucleotide composition is: A of 40.7%, T of 40.3%, C of 11.3% and G of 7.7%, with a total GC content of the mitochondrial genome 19.0% and AT of 81.0%. Phylogenetic and taxonomic analysis based on the amino acid sequences of 13 PCGs from 21 species confirmed the position of *S. frugiperda* closely related to *S. litura* and *S. exigua*. The complete mitochondrial genome of *S. frugiperda* study is able to provide a reference for the phylogenetic and taxonomic relationships, and also more data for further study of the Nolidae family in China.

*Spodoptera frugiperda* is a worldwide pest of economic importance for different crops, such as maize, cotton, rice sorghum, and millet. *Spodoptera frugiperda* is also known as fall armyworm and is a species native to the American tropical and subtropical regions of the polyphagous pest (Sparks 1979; Todde and Poole 1980). From January 2016 to July 2018, *S. frugiperda* has invaded African and some Asian countries, and it was also found in Yunnan of China in early 2019 (Nagoshi et al. 2018). Now, there are few studies on *S. frugiperda* genomics, also little knowledge about the mitochondrial genome of *S. frugiperda* and other Nolidae family insects is available. So, in this research, we assembled and annotated the complete mitochondrial genome of *S. frugiperda* and discussed the phylogenetic and taxonomic relationship with other 21 species, which contributes to the study of the insect of *S. frugiperda* and provides more data for the further study of the Nolidae family in China.

The specimen sample of *S. frugiperda* was collected from Yu ling (Yuling, Shaanxi, China, 109.77E; 38.30N). The whole genomic DNA was purified and fragmented using g-TUBES (Covaris, Inc., Woburn, MA) and the genome sequences of *S. frugiperda* (GenBank NJHR00000000.1) were used to assemble the mitochondrial genome, which was stored in Yulin University College of Life Science (YLU002). Quality reads and adapters control was performed and low-quality reads were removed, using the NGS QC Toolkit software (Patel and Jain 2012). The mitochondrial genome was assembled and annotated using the MitoZ software (Meng et al. 2019). The physical map of the new mitochondrial genome was generated using OGDRAW (Lohse et al. 2013).

The complete mitochondrial genome of *S. frugiperda* (GenBank accession No. MN094786) was a closed-circle with 15,353 bp in size, which is well within the size range observed in the completely sequenced mitogenomes. The mtDNA of *S. frugiperda* comprised 37 genes, including 13 protein-coding genes (PCG) (*ND1-6*, *ND4L*, *ATP6*, *ATP8*, *COX1-3* and *CYTB*), 22 transfer RNA genes (tRNA), 2 ribosomal RNA genes (rRNA) (*12S rrn* and *16S rrn*), and 1 D-loop control regions. The overall nucleotide composition is: 40.7% A, 40.3% T, 11.3% C, and 7.7% G, with a total GC content of 19.0% and AT of 81.0%.

Phylogenetic and taxonomic analysis was done using the maximum-likelihood (ML) methods, we selected and analyzed the relationship of other 20 species using the mitochondrial genomes of 13 PCGs with *S. frugiperda*. Maximum-likelihood (ML) methods analysis was performed using RAxML software (Stamatakis 2014) with the GTR + G + I model, and using the number of bootstrap replicates as 5000. Phylogenetic relationship obtained with the ML approach was identical to those obtained using the Bayesian analysis (BI). The Bayesian phylogenetic alignment was analyzed using MrBayes version 3.2.5 software (Ronquist and Huelsenbeck 2003) based on the most appropriate model. The phylogenetic tree was represented using MEGA X (Kumar et al. 2018) and edited using FigTree version 1.4.4. The phylogenetic ML tree based on the putative amino acids sequences from all 13 PCGs excluding the stop codons are shown in Figure 1, the mitochondrial genome of *S. baicalensis* is clustered and closest to *Spodoptera litura* (GenBank accession No. NC_022676.1) and *Spodoptera exigua* (GenBank accession No. NC_019622.1) in the genetic evolutionary relationship.

**Figure 1.**
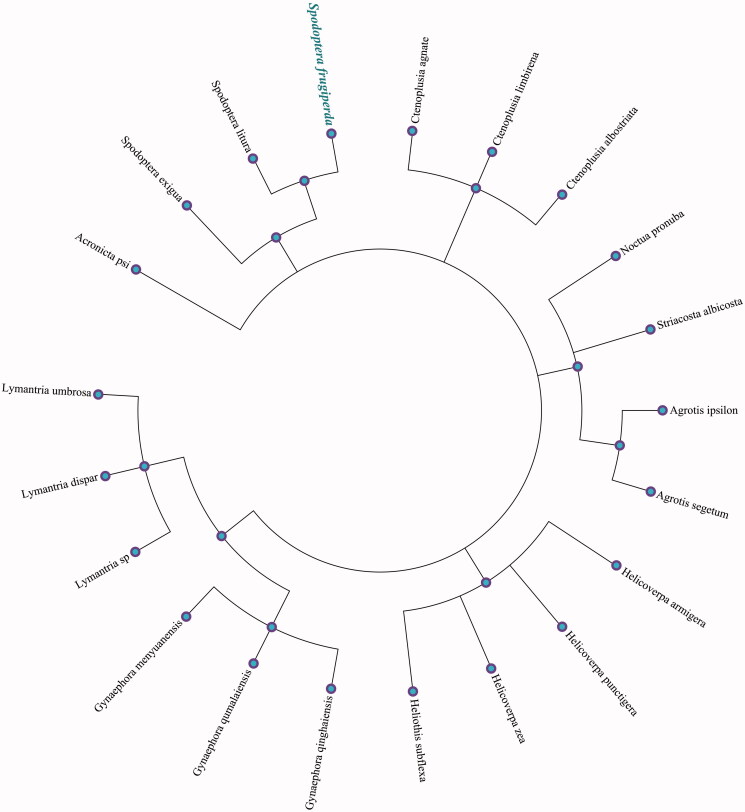
The maximum-likelihood (ML) phylogenetic tree from 21 species mitochondrial genomes using 13 PCGs. Numbers above each node indicates the ML bootstrap support values. 20 species accession numbers in this study have been deposited in the GenBank are as follows: *Acronicta psi* (KJ508060.1)*, Agrotis ipsilon* (NC_022185.1)*, Agrotis segetum* (NC_022689.1)*, Ctenoplusia albostriata* (HQ951244.1)*, Ctenoplusia agnate* (NC_021410.1)*, Ctenoplusia limbirena* (NC_025760.1)*, Gynaephora menyuanensis* (NC_020342.1)*, Gynaephora qinghaiensis* (NC_029163.1)*, Gynaephora qumalaiensis* (NC_029164.1)*, Helicoverpa armigera* (NC_014668.1)*, Helicoverpa punctigera* (NC_023791.1)*, Helicoverpa zea* (NC_030370.1)*, Heliothis subflexa* (NC_028539.1)*, Lymantria dispar* (NC_012893.1)*, Lymantria sp* (KY923068.1)*, Lymantria umbrosa* (NC_035627.1)*, Noctua pronuba* (KJ508057.1)*, Spodoptera litura* (NC_022676.1)*, Spodoptera exigua* (NC_019622.1)*, Striacosta albicosta* (NC_025774.1).
